# Novel image registration approach for combining 2D Osterix and collagen bundles images with 3D micro-CT

**DOI:** 10.1093/jbmrpl/ziag009

**Published:** 2026-01-28

**Authors:** Mireille Ngokingha Tchouto, Julia Mehl, Saeed Khomeijani Farahani, Daniel Baum, Georg N Duda

**Affiliations:** Julius Wolff Institute, Berlin Institute of Health at Charité, Universitätsmedizin Berlin, 13353 Berlin, Germany; Berlin Institute of Health at Charité, Universitätsmedizin Berlin, Core Unit Bioinformatics, 10117 Berlin, Germany; Berlin-Brandenburg School for Regenerative Therapies, Charité, Universitätsmedizin Berlin, Corporate Member of Freie Universität Berlin and Humboldt-Universität zu Berlin, 13353 Berlin, Germany; Julius Wolff Institute, Berlin Institute of Health at Charité, Universitätsmedizin Berlin, 13353 Berlin, Germany; BIH Center for Regenerative Therapies, Berlin Institute of Health at Charité, Universitätsmedizin Berlin, 13353 Berlin, Germany; Julius Wolff Institute, Berlin Institute of Health at Charité, Universitätsmedizin Berlin, 13353 Berlin, Germany; BIH Center for Regenerative Therapies, Berlin Institute of Health at Charité, Universitätsmedizin Berlin, 13353 Berlin, Germany; Zuse Institute Berlin, Visual and Data-Centric Computing, 14195 Berlin, Germany; Julius Wolff Institute, Berlin Institute of Health at Charité, Universitätsmedizin Berlin, 13353 Berlin, Germany; BIH Center for Regenerative Therapies, Berlin Institute of Health at Charité, Universitätsmedizin Berlin, 13353 Berlin, Germany

**Keywords:** 2D-3D registration, bone healing, μCT, SHG, immuno-histochemistry, mechano-sensation

## Abstract

To study bone healing, different imaging techniques are typically employed. Histological and immunohistological methods allow the visualization of Osterix (OSX) expression and collagen structures, whereas μCT imaging enables the assessment of mineralized tissue. However, a clear spatial alignment between the information obtained from these 2 modalities is still lacking. In this study, we present a technological approach for registering 2D histological sections of collagen bundles and OSX signals with 3D volumetric μCT data. We applied this method to datasets from animals with rigid and semi-rigid fracture fixation to resemble fast and effective healing and delayed healing. Using our 2D-3D registration workflow, we were able to identify corresponding 2D μCT and histological slices, and we showed that the algorithm performed consistently across both fixation conditions, resembling the healing consequences. Thus, we could illustrate how such image registration techniques could be used to study the co-localization of OSX, collagen, and hydroxyapatite (HA). This framework enables the visualization and direct comparison of OSX, collagen, and HA within a single, spatially matched image, providing a tool for future studies to quantitatively explore tissue co-localization and spatial relationships during bone healing.

## Introduction

Image registration is the process of adjusting images to co-align accurately enough using fixed reference information in space and time.^1^ Such co-alignments have broad applications across the medical domain, enabling consistent comparison and integration of different imaging modalities.^2^ Achieving a complete diagnostic view often requires the use of multiple imaging technologies, each capable of highlighting different aspects of the underlying structures. Among such modalities are second harmonic generation (SHG) imaging and micro-CT (μCT), which provide complementary information about the sample.

Second harmonic generation is a powerful method for visualizing thick collagen structures and their organization^3^^,^^4^ and it allows to generate 2D images of collagen bundles with high resolution. To date, μCT is the gold standard method for quantifying BMD and bone microstructure in a 3D space.^5–7^ However, μCT does not allow to visualize soft tissues, except if contrast agents would be used.^5^^,^^8^ Due to the difference in spatial resolution, these 2 methods are applied separately within a study, and their results are interpreted indirectly and not directly by matching their structures one-to-one.^5^ However, by directly combining their information, the insights gained from the respective information could be reinforced.

The 2D-3D registration is a method for aligning 2D images such as histological section images to 3D volumetric information such as image stacks.^6^^,^^9^^,^^10^ The difficulty of this method is finding the 2D slice in a 3D volume and correcting for the deformation present in the histology image due to the slicing process during sample preparation.^8^^,^^11^ Many registration methods have been developed to perform such registration.^5^^,^^12–14^ One of such was that of Hoerth et al.,^12^ where the generalized Hough transform (GHT) was used to find the shape of 2D backscattered electron images in 3D μCT images. This method was only applied to electron microscopy images, which have a similar resolution to μCT images and visualize structures that are almost identical to those of μCT images. In addition, the distortions occurring in histology images were not considered and the method was not tested on histology images, thereby underscoring the need to apply these methods to high-resolution histology images.

In this study, we adapted the registration framework proposed by Hoerth et al.^12^ to enable the alignment of 2D SHG and Osterix (OSX)-stained images with corresponding 3D μCT datasets. Additionally, we presented an illustrative example to highlight the applicability and potential benefits of this multimodal integration.

## Materials and methods

### Data used

The data used in this study were taken from our previously published study on the effect of external mechanical stability on the regulation of hematoma vascularization during bone healing.^15^

Cdh5-iCreERT2 female mice, aged 10 wk at the time of surgery, were used in this study.^15^ The animals were randomly distributed across the rigid and semi-rigid treatment groups. Surgical procedures were performed at the Charité animal facility, and femurs were collected 7 or 14 d post-operation (LaGeSo G0322/18).^15^ To prepare a mouse osteotomy model for studying the mineralization phase of bone healing, animals were anesthetized using isoflurane administered via a face mask delivering an isoflurane-oxygen mixture. While under anesthesia, the animals received a subcutaneous injection of buprenorphine (Temgesic, 0.03 mg/kg) for preoperative analgesia. To prevent infection during surgery, prophylactic antibiotic treatment with clindamycin (8 mg/kg) was administered subcutaneously, also under anesthesia. Throughout the procedure, mice were placed on a warming plate to prevent hypothermia. In addition, eye ointment was applied to protect the cornea from drying.^15^

An external fixator (MouseExFix 100% and 50%, RISystem) was mounted on the left femur. The device consisted of 4 screws (Ø = 0.45 mm) and a radiolucent fixator body. A longitudinal lateral skin incision was made from the knee to the hip joint to access the femur, taking care to preserve the sciatic nerve. The iliotibial tract and vastus lateralis muscle were dissected to expose the bone. Using a hand-held drill, the first pinhole was created just proximal to the distal metaphysis of the femur. Three additional pinholes were then drilled to complete fixation of the external fixator. Once the fixator was aligned parallel to the femoral axis, a 0.7 mm osteotomy was performed between the 2 central pins using a wire saw (RISystem, Davos, Switzerland). The surgical wound was then closed in layers. Postoperative analgesia was provided by administering tramadol (25 mg/L) via the drinking water for 3 consecutive days. During the healing period, animals were able to move freely in their cages with the fixator in place. At the study endpoints—7 and 14 d post-osteotomy—the animals were euthanized under deep anesthesia using a ketamine-medetomidine mixture, followed by cervical dislocation.^15^ The femurs of the mice were collected and incubated in 4% paraformaldehyde in PBS for 6-8 h on a shaker. Osterix staining was performed as previously described.^15^ To visualize the OSX-stained cryosections, a Leica SP8 confocal microscope was used. To visualize fibrillar collagen, SHG imaging was performed using a multiphoton laser with an excitation wavelength of 910 nm. Z-stacks were acquired for each image. For the visualization of hydroxyapatite (HA), samples were scanned using a Bruker SkyScan 1172 high-resolution micro-CT (μCT) system (Bruker, Kontich, Belgium).^15^

To evaluate our registration framework, we used data from the control group at 14 d post-surgery, as μCT scans were only available at this time point. In total, we included 10 samples in our study: 5 from the semi-rigid group (resulting in delayed bone healing) and 5 from the rigid group (resulting in fast and effective healing).

### 2D-3D registration workflow

A previously developed framework in Amira (Thermo Fisher Scientific)^16^ was used for the 2D-3D registration workflow.^12^ Only a few adjustments were necessary to adapt it to the datasets of this study. Since the system relied on the GHT to find the shape of the 2D image in the 3D μCT, templates had to be created for both the 2D image and the 3D image. A similarity between the 2 images had to be found, and since the SHG image, like the μCT image, has cortices, it was used as the moving image. From the Z-stack of SHG images, the maximum projection was estimated and used for registration. To speed up the calculation, both the SHG image and the μCT images were downsampled. For the 2D image, the template was created by segmenting the image and extracting only the cortices and calculating landmarks around the cortices. This made it easier to find the shape in the 3D μCT image. For the 3D μCT, the template was created by calculating the gradient image to facilitate edge detection.

The GHT was then calculated by inputting the 3D μCT image and the 2D image as well as the created templates. In this way, the 2D μCT slice corresponding to the cutting plane of the SHG image was found. This first registration result was then further refined using the AffineRegistration module of Amira. To extract the found 2D μCT slice, the Amira slicing module Oblique Slice was used. For further precision, the 3D image was manually rotated to obtain the exact plane. Then, the 2D image was extracted and resized to the size of the full 2D SHG image. Since the OSX and SHG images came from the same device, it was easy to apply the transformation obtained from the SHG image to the full-size OSX image.

Finally, to correct the distortion in the histology images caused by sectioning during sample preparation,^9^^,^^11^ a 2D-2D Elastix image registration was performed between the found 2D μCT section and the 2D SHG image. The ITK library^17^ in Python was used for this purpose.^18^ Finally, registration metrics were calculated, such as the Dice score (DSC) to evaluate the accuracy of the registration fit,^19^ the Jacobian determinant (J) to evaluate the degree of deformation in the histology images,^20–22^ and the structural similarity index (SSIM) to evaluate the similarity between structures in the μCT and SHG^21^. Segmented images were used for the estimation of DSC and SSIM to compensate for the large structural differences between μCT and SHG images. To better approximate the DSC and SSIM, the images were divided into 2 parts: the proximal and distal parts. The gap was omitted. This was because we were primarily interested in the alignment of the cortices, as these structures were similar in both images. The final scores for the DSC and SSIM were estimated as follows:


$$ \mathrm{DSC}=\left(\mathrm{DS}{\mathrm{C}}_{\mathrm{proximal}}+\mathrm{DS}{\mathrm{C}}_{\mathrm{distal}}\right)/2 $$

$$ \mathrm{SSIM}=\left(\mathrm{SSI}{\mathrm{M}}_{\mathrm{proximal}}+\mathrm{SSI}{\mathrm{M}}_{\mathrm{distal}}\right)/2 $$

The entire raw image was used to estimate the extent of distortion in the SHG image compared to the 2D μCT image, as this was more suitable for a better assessment of the deformation field. The ITK library in Python was used for this purpose. The Skimage^23^ metric was used for the estimation of the SSIM.

### Example of application: proximity analysis

The maximum projection image could not be used for the proximity analysis because the neighborhood of the cells needed to be characterized. For this purpose, the Laplacian function was used to find the best-focused image of the 2D Z-stack of the OSX images. The same was done for the SHG image to have the same focal plane for both. The images were then re-registered using the stored transformation obtained from the original registration between the maximum projected SHG image and the 3D μCT image. This was not a problem because the registration focused on the image edges, namely the cortices, rather than the structures in the BM. Therefore, the maximum projected image was best suited for better registration, as the edges were most visible there. After re-registering the images, a neighborhood analysis was performed. First, the positive signal for each of the images—namely, the corresponding 2D μCT image, the SHG image, and the OSX image was determined using the Otsu thresholding^24^ method. These positive signals or positive cells represent areas in which at least 1 expressing cell was present. The coordinates of these positive cells were then determined, and the Euclidean distance^25^ between the coordinates of 1 image and the coordinates of the other image was estimated. Based on these coordinates, the minimum distance, which represents the smallest distance between the structures, was determined. Distances were estimated only for positive cells. To avoid a memory shortage, the proximity analysis was not computed directly on the whole image. The workaround was to divide each image into smaller blocks, estimate the proximity analysis in each of the blocks, and store the distances estimated in all blocks in a single vector.

### Statistical analyses

The Wilcoxon rank-sum test was performed using the scipy (v1.7.3) package within an Anaconda^26^ (v2.1.4) environment running Python 3.9 to compare the means between 2 groups. Statistical significance was defined as ^*^p < .05, ^**^p < .01, and ^****^p < .0001.

## Results

### 2D-3D registration finds corresponding 2D μCT slice of SHG image

**Figure 1:**
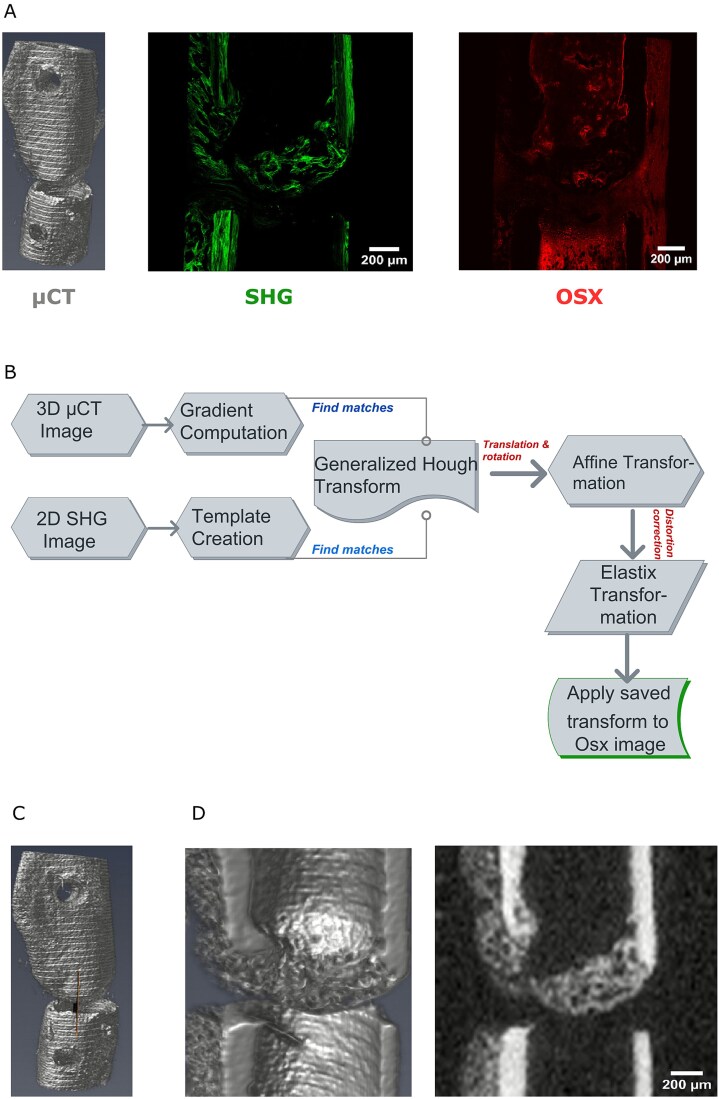
2D/3D registration finds corresponding 2D μCT slice of SHG image. (A) Images used in this study: on the left, the 3D μCT image; in the center, the 2D SHG image used as the registration template; on the right, the 2D Osterix image. (B) Schematic representation of the registration procedure. (C) Position of the 2D image found in the 3D μCT volume. (D) Located μCT image corresponding to the 2D SHG image: on the left, the μCT image as 3D volume; on the right, the μCT image as 2D slice.

We sought to integrate 3D μCT volumes with 2D high-resolution histology images to enable multimodal analysis. To this end, we used the following published data consisting of OSX, collagen, and μCT images^15^ (Figure 1A). To directly match the different structures one-to-one, we expanded the previously published registration framework^12^ (Figure 1B) (see “Methods” for details). With the registration, we were able to find the position of the 2D SHG image in the 3D μCT volume (Figure 1C), slice the 3D image at this cutting plane (Figure 1D, left) and extract the 2D μCT slice corresponding to the same cutting plane as the 2D SHG image (Figure 1D). The extracted 2D μCT slices corresponding to the same cutting plane as the 2D SHG image for all 10 samples is shown in Figure S1.

### No significant difference in the registration metrics between rigid and semi-rigid

**Figure 2:**
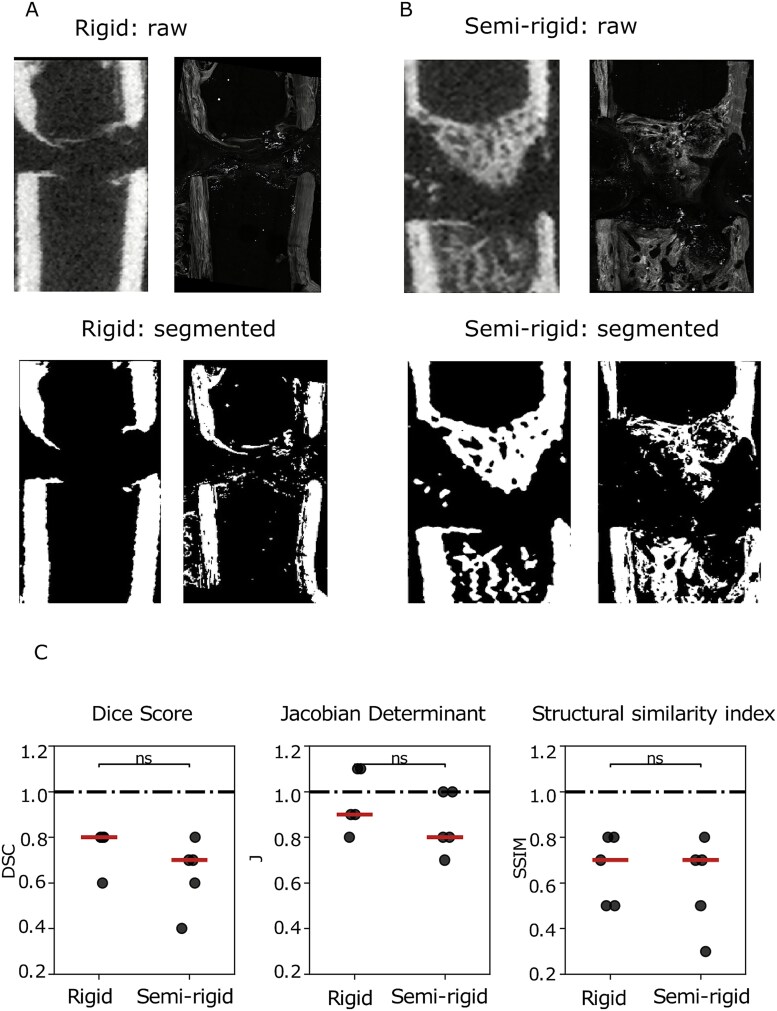
No significant difference in the registration metrics between rigid and semi-rigid. (A and B) 2D μCT image and corresponding 2D SHG image with their corresponding segmented images in rigid (A) or semi-rigid (B). (C) Boxplot illustrating the estimated registration metrics namely dice score, Jacobian determinant and structural similarity index. Significant differences in registration metrics were evaluated using Wilcoxon rank sum test and are indicated with an asterisk (ns: not significant).

Next, we wanted to evaluate the accuracy of our registration workflow. To do so, we calculated the DSC, the Jacobian determinant, and the SSIM (see “Methods”) between the SHG image and the corresponding 2D μCT image for each animal and compared these values between the rigid and semi-rigid groups (Figure 2A and B). Overall, we found no significant differences in the registration metrics between the semi-rigid and rigid fixations (Figure 2C).

Due to the extreme differences in structure and resolution between the μCT images and SHG images, it was difficult to obtain a Dice score of 1 or an SSIM of 1 even for our best-aligned image. For our best-aligned image, we obtained a DSC of 0.8 (Figure 2C). In summary, despite the large differences in resolution between μCT and SHG, our registration workflow was able to find the 2D μCT slice corresponding to the 2D SHG image, and the registration metrics showed that the detected slices were quite similar.

### Example of application: proximity analysis of OSX and collagen relative to μCT in different fixation groups

**Figure 3:**
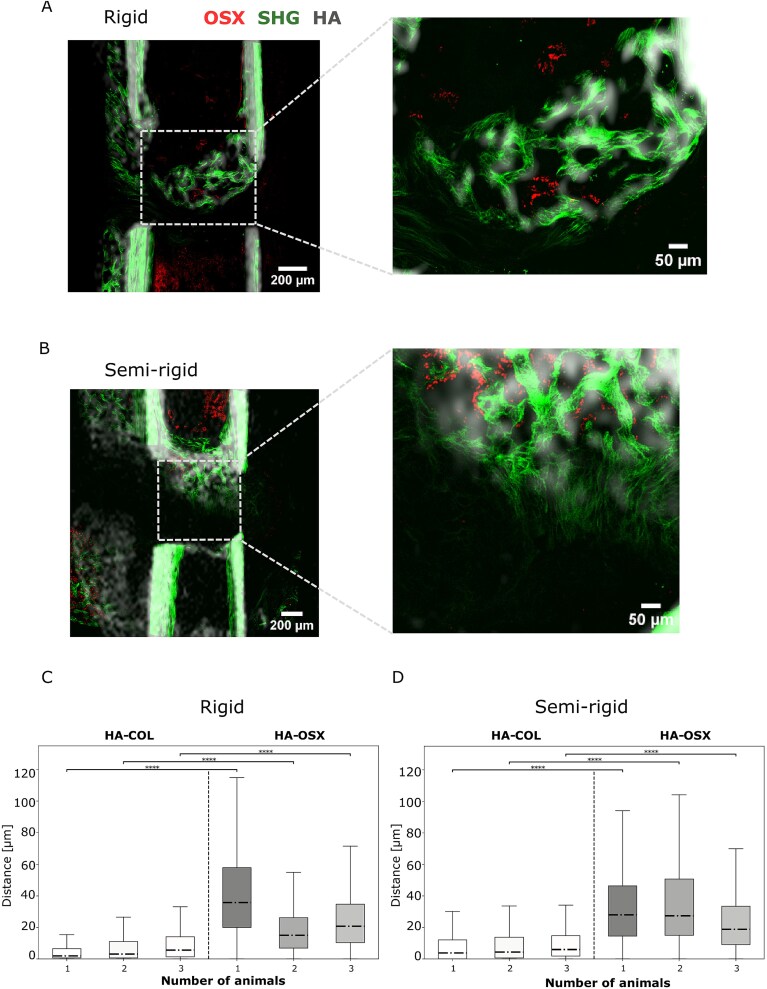
Greater distance between Osterix (OSX) and hydroxyapatite (HA) than between HA and collagen. (A and B) Zoom in of the fracture gap of the merged images of 2D μCT image, the SHG and OSX in rigid (A) or semi-rigid (B). (C and D) Boxplots showing the proximity analysis in the fracture gap between HA and SHG and HA and OSX in rigid (C) or semi-rigid (D) (*N* = 3 per group). Wilcoxon rank sum test was used for comparisons. The lower and upper hinges of box plots correspond to the 25th and 75th percentiles, respectively. Center lines of box plots depict the median values. Significant differences are indicated with asterisks (^****^p < .0001).

**Figure 4:**
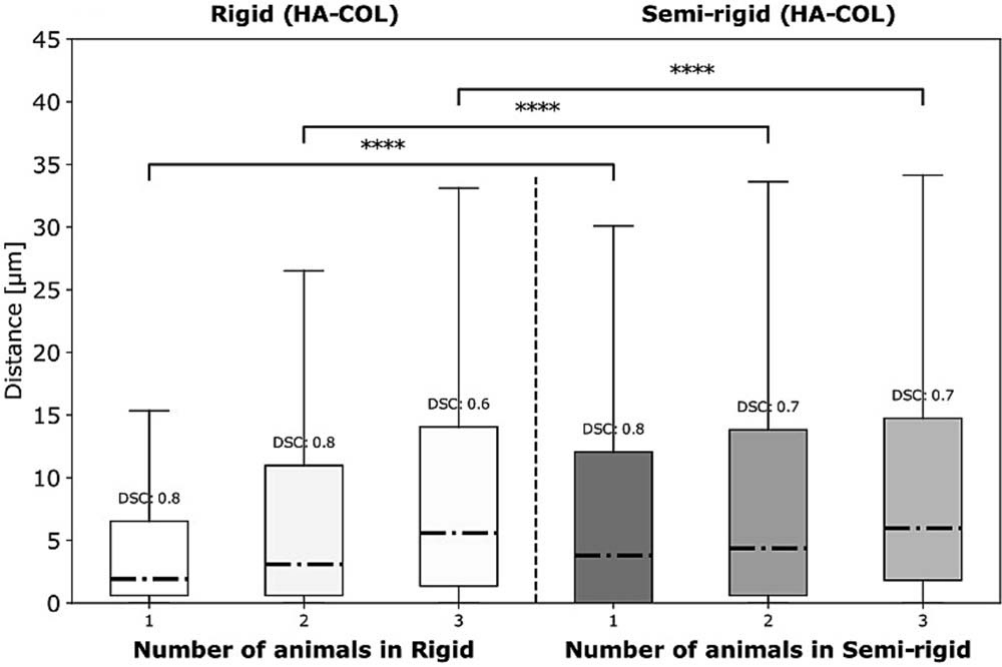
Quantification of HA–collagen proximity across fixation conditions. Boxplots showing the proximity analysis between HA and collagen in rigid and semi-rigid (*N* = 3 per group). Wilcoxon rank sum test was used for comparisons. The lower and upper hinges of box plots correspond to the 25th and 75th percentiles, respectively. Center lines of box plots depict the median values. Significant differences are indicated with asterisks (^****^p < .0001).

One application of this multimodal registration framework is to study the spatial distances between OSX and HA, as well as between collagen and HA. To achieve this, we overlaid the extracted 2D μCT slices with the corresponding SHG and OSX images (Figure 3A and B) and applied our proximity analysis method to estimate the nearest distances between the structures within the fracture gap. This analysis was performed on the 3 highest-quality images from each of the rigid and semi-rigid groups, selected to ensure consistent representation between the 2 fixation conditions. These preliminary results indicate that, in both fixation groups, the distance between HA and OSX was generally larger than the distance between collagen and HA (Figure 3C and D). Furthermore, the framework also allowed us to directly compare the distances between HA and collagen across the rigid and semi-rigid groups (Figure 4), revealing a trend toward shorter HA-collagen distances in the rigid group compared to the semi-rigid group. Altogether, these findings demonstrate the potential of 2D-3D registration to provide insights into the spatial co-localization of tissues during bone healing.

## Discussion

In this study, we used a novel image-matching approach to register high-resolution 2D histology images into 3D μCT volumes. To achieve this, we integrated previously published data on collagen bundles, 2D OSX-stained histological sections, and 3D volumetric μCT data.^15^ The novelty of our work lies in the simultaneous registration of both the 2D SHG and 2D OSX images into the 3D μCT dataset, enabling a comprehensive characterization of the spatial co-localization between OSX, collagen, and HA within a unified imaging framework.

Several methods have been developed for the registration of 2D slices in 3D datasets.^27–29^ One of them is that of Hoerth et al.^12^ who developed a semi-automatic framework for the registration of 2D slices in 3D volumes using the GHT. The original system did not consider the distortions in the 2D images, as it was only tested on electron microscopic images. In this study, we were able to extend the system by adding the Elastix transformation so that it can also be used for histology images. Despite the large differences in resolution between the μCT (10.5 μm) and SHG (0.6 μm) data, as well as the distinct structures visualized by each modality, our registration framework was able to find the corresponding 2D μCT slices from the 2D SHG images. Nevertheless, there were cases in which the images could not be perfectly aligned, despite the correct 2D μCT slice being detected. Future studies could refine the proposed framework to better compensate for this issue.

Moreover, although we did not find statistically significant differences in key registration metrics—such as the DSC, Jacobian determinant, and SSIM between the groups—we observed a trend toward slightly better values in the rigid fixation group. While the underlying cause of this trend remains uncertain, we speculate that improved cortical delineation in the rigid samples may have contributed. Overall, we successfully adapted and optimized the existing registration framework for application to histological images. Future studies with larger sample sizes in both the rigid and semi-rigid fixation groups will be essential to confirm the consistency of the observed trends, clarify their underlying mechanisms, and further evaluate the robustness of the registration workflow.

Osterix is a transcription factor essential for osteoblast differentiation and the production of type I collagen (Col1).^30^^,^^31^ Collagen secreted by osteoblasts also provides the structural template on which mineral deposition occurs.^4^^,^^32^ Using our 2D-3D registration method, we were able to integrate these different structures—OSX, collagen, and HA—into a single, spatially aligned image. However, due to the limited sample size, we were unable to analyze this bone mineralization sequence in detail. Future studies with larger cohorts could investigate the spatial relationships between OSX, collagen, and HA in greater detail, enabling a clearer understanding of the sequential steps of bone mineralization during healing and how these processes vary under different mechanical fixation conditions.

Beyond its application in preclinical research, the multimodal registration framework developed in this study may also hold potential for future clinical imaging and patient-specific monitoring of bone healing. 2D-3D registration techniques are already used in surgical navigation and preoperative planning, especially for the real-time alignment of intraoperative 2D X-ray images with preoperative 3D scans.^2^ In a similar way, the 2D-3D registration method used in our work could be applied to monitor bone-healing progression in a more personalized manner. By assessing the spatial relationships between collagen, HA, and OSX, and relating these to the bone volume detected through radiographic scans, this approach could help clinicians evaluate healing status in real time. Nevertheless, several challenges must be addressed before such an approach can be translated into clinical practice. Replicating this level of detail in humans would require advanced imaging infrastructure, increased cost, and potential exposure to higher radiation doses.^33^ Moreover, implementing 2D-3D registration in clinical workflows would require substantial automation and regulatory approval. Although further work is needed for clinical implementation, our approach illustrates the potential of combining histological and volumetric imaging to better capture the spatial dynamics of bone healing.

In summary, we proposed a method to integrate high-resolution OSX and collagen images with 3D μCT volume data. Future work may further optimize this registration framework to enable the detailed quantification of spatial interactions between collagen, OSX, and HA, thereby advancing our understanding of mineralization dynamics during bone healing.

## Supplementary Material

Supplementary_material_figure_captions_ziag009

## Data Availability

The data are available upon demand.
