# Supplementary material for: Novel image registration approach for combining 2D Osterix and collagen bundles images with 3D micro-CT
Source: JBMR Plus. 2026 Jan 28;10(3):ziag009. doi: 10.1093/jbmrpl/ziag009 (PMC12906291; doi:10.1093/jbmrpl/ziag009)

**SFig.1** Display of all images used in this study. **a)** Rigid 2D images: top row, 2D SHG image; bottom row, corresponding 2D µCT image. **b)** Semi-rigid 2D images: top row, 2D SHG image; bottom row, corresponding 2D µCT image.


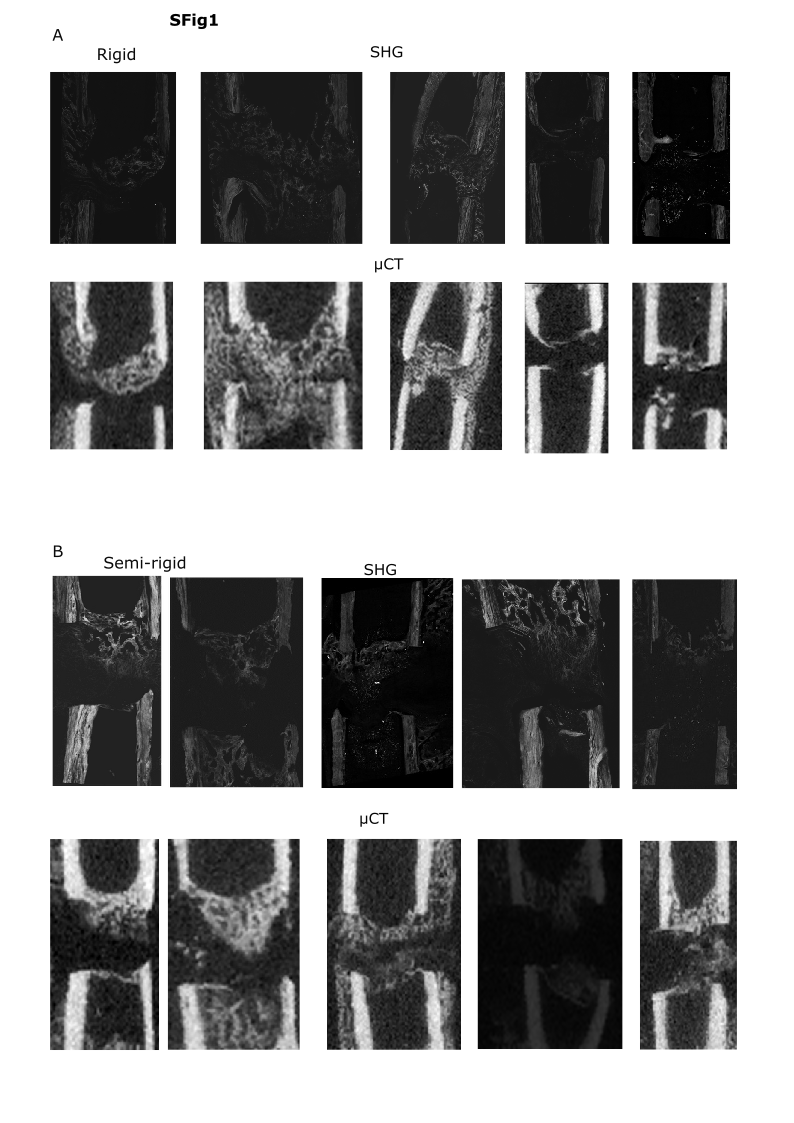

Supplement: Supplementary_material_figure_captions_ziag009 [file supplementary_material_figure_captions_ziag009.docx]
